# 

**Published:** 1918-08-24

**Authors:** 


RATES OF SUBSCRIPTION:

Twelve months, 10/10; Six months, 5/5; Three months, 2/9, post free to any address in the United Kingdom. Abroad, Twelve months, 13/-; Six months, 6/6; Three months, 3/3, post free. **THE HOSPITAL "Index" to Volume LXI., October 19 7 to March 1918, is now ready, price 3½d. per copy, post free.** Address The Manager, The Hospital, "The Hospital" Building, 28/29 Southampton Street, Strand, London, W.C. 2.

SIR GEORGE HARE PHILIPSON, M.D., President of Durham College of Medicine and Newcastle Royal Victoria Infirmary, left £2,000 to the University of Durham to found medical scholarships, £1,000 to Newcastle Royal Infirmary Chaplaincy Endowment Fund, and £1,000 as a fund for the payment of the income of a chaplain of the Prudhoe Memorial Convalescent Home, Whitley Bay.

The Cardigan County Council have appointed Dr. Meredith Davies, of Carmarthen, to be Medical Officer of Health for the shire. Dr. Davies, who is under thirty years of age, had a brilliant school career. He was lately discharged from the Army, in which he held captain's rank, and was awarded the Mons ribbon. Upon his election one of the councillors observed that he hoped their new medical officer would wear the ribbon in order to remind them of the gallantry at Mons. The salary attached to Dr. Davies' new post is £500 per annum, with £120 for travelling expenses.

MISS GRACE CADELL, L.R.C.P., L.R.C.S., of Edinburgh, left £2,000 to the Edinburgh Hospital for Women and Children to endow a bed in the maternity department in memory of her mother, and £1,000 to the Queen's Nurses in Scotland. The residue of the property was left equally to the University of Edinburgh, to help in the medical education of women, and the Edinburgh Hospital for Women and Children.

### Matrons' and Sisters' Appointments.

Auxiliary Military Hospital, Poulton-le-Fylde.—Sister G. Edmonds has been appointed sister-in-charge. She was trained at Huntingdon County Hospital, and has been matron of Bridlington V.A.D. Hospital.

Disabled Sailors' and Soldiers' Home, Southport.—Miss Catherine Doughty, A.R.R.C., has been appointed matron. She was trained at the Royal Victoria Hospital, Newcastle-on-Tyne, and has since been sister at Norfolk and Norwich Hospital.

Freemasons' War Hospital, S.W.—Miss Hetty Burn has been appointed sister. She was trained at the Derbyshire Royal Infirmary; has been sister at the Royal Sea-bathing Infirmary, Margate; St. Mark's Hospital, City Road; Hampstead General Hospital; and Miller General Hospital, Greenwich; and pupil-housekeeper at the Hospital for Sick Children, Great Ormond Street.

Pate Auxiliary Hospital, Llandderfel, Corwen.—Miss M. Pineo has been appointed alternate night and massage sister. She was trained at the Royal Hampshire County Hospital, Winchester, and since conducting a nursing home at Bournemouth has been night sister at Harborne Hall Auxiliary Hospital, Birmingham, and sister-in-charge at the Lindfield Red Cross Hospital.

Perth County and City Royal Infirmary.—Miss Barbara McLean has been appointed night sister. She was trained at Oldham Royal Infirmary, and has since been sister at the Cameron Hospital, West Hartlepool, sister of the x-ray and out-patients' department at Bolton Infirmary, and sister of the male wards at the Royal Salop Infirmary, Shrewsbury.

Workmen's Hospital, Oakdale, Mon.—Miss Margaret Ellis has been appointed matron. She was trained at Cardiff Union Hospital.

**NOTE.—Particulars of Appointments for insertion in this Column will be welcomed.**

### Recent Official Publications.

(To be obtained from H.M. Stationery Office, Imperial House, Kingsway, W.C. 2.)

The Statistics relating to National Health Insurance and Regulations Affecting the Administration of Approved Societies. Post free 3s.

Reports of the Local Government Board on Public Health and Medical Subjects (New Series, No. 117). The Incidence of Small-Pox throughout the World in Recent Years, by Dr. R. Bruce Low. Post free 2s. 2d.

Reports of the Local Government Board on Public Health and Medical Subjects (New Series, No. 119). Reports and Papers on Malaria Contracted in England in 1917. Post free 4s. 3d.

Midwives Bill. Memorandum. Post free 3d.

National Health Insurance Medical Research Committee. Report upon the Seasonal Outbreak of Cerebro-Spinal Fever in the Navy at Portsmouth, 1916-17. Report upon the Treatment of Cerebro-Spinal Meningitis by Anti-meningococcus Serum at the Royal Naval Hospital, Haslar, 1915-16-17. Post free 2s. 9d.

Workmen's Compensation (Silicosis) Act, 1918. Ch. 24. Post free 1½d.

### Ministry of Health.

It is expected that a Government Bill will be introduced at the beginning of the coming Parliamentary Session.

### Organising a Rural Appeal.

LADIES' WORK IN SUSSEX.

Attention must be drawn to the result of an effort of a band of ladies in the county of Sussex on behalf of the Royal Sussex County Hospital. Earl Brassey, the president, issued early in the year an appeal for £10,000 to tide over 1918. The collection of the last £2,000 or so is causing some anxiety. However, Mrs. Lea and Mrs. Frank Wisden made an appeal, either for cash or the proceeds of the sale of goods, in a large rural area of the county. Divided into twenty-six districts, with a lady in charge of each—save one where the vicar officiated—each district was asked to subscribe by a certain date. The result was that the twenty-six districts contributed £701. A glance at the list of the districts shows that a good slice of the county of Sussex was included in the scheme. If the remaining parts are similarly dealt with, £10,000 should be speedily realised.

### Difficulties of the School Medical Inspection.

Authorities are feeling the effects of the shortage of doctors and nurses. The annual report of the Medical Superintendent of Schools under the Lancashire Education Committee, for example, declares that the work of medical inspection had been carried on under difficulties, which tend to increase. Now the authority has eight doctors and ten nurses on war service, and the report says the depletion of the ordinary civil members of the profession has been so extensive that it has been found impossible to make any temporary appointments to the staff. The work of school medical inspection is now recognised to demand a varied standard of attainment not only in general medicine. The available number of medical men possessed of the required versatility and experience is at present very limited. Moreover, the marked deterioration in the railway service has made the work of medical officers and nurses much more arduous than formerly, whilst the increased fares have to an extent too little realised made it impossible for parents to obtain specialised treatment for their children in the hospitals, which are often distant from their homes.

### The Book Market.

LIST OF NEW BOOKS RECEIVED FROM THE FOLLOWING PUBLISHERS:—

J. B. Lippincott Co.: "First-Aid in Emergencies." By Eldridge L. Eliason, M.D. 6s. net.

T. Fisher Unwin, Ltd.: "Baby Welfare: A Guide." By W. E. Robinson, M.D. 7s. 6d. net.

J. and A. Churchill: "Massage and Medical Gymnastics." By Dr. Emil A. G. Kleen. 21s. net.

John Wright and Sons, Ltd.: "The British Journal of Surgery." April 1918. 8s. 6d. net. "The Medical Council, 1918." 10s. net. "War Wounds of the Lung." By Pierre Duval. 8s. 6d. net.

Longmans, Green and Co.: "Anatomy, Descriptive and Applied." By Henry Gray, F.R.S., F.R.C.S. Twentieth Edition. Edited by Robert Howden, M.A., D.Sc., M.B., C.M. 37s. 6d. net.

Charles Griffin and Co., Limited: "Food and Dietetics." A Manual of Clinical Dietetics. By Sir R. W. Burnet, K.C.V.O., M.D., J.P.

